# Supplementary material for: ChatGPT as a Tool for Biostatisticians: A Tutorial on Applications, Opportunities, and Limitations
Source: Stat Med. 2025 Oct 23;44(23-24):e70263. doi: 10.1002/sim.70263 (PMC12548020; doi:10.1002/sim.70263)
Supplement: Supplementary file 1 — Data S1. Supporting Information. [file SIM-44-0-s001.zip › Supplementary Materials Additional.zip!/3.3_Individual-Level_Surrogacy/3.3_Surrogacy_Chat2.pdf]

Information Theory and Surrogacy

Can you explain me the information theoretic approach to individual-level surrogacy? Please cite me a paper that treats the subject from the predictive angle.

Can you explain to me the difference between the meta-analytic approach and the information theoretic approach to individual level surrogacy?

What is mutual information?

Is there a relationship between mutual information and the deviance?

What is the Likelihood Reduction Factor and how does it relate to mutual information?

Can you help me to calculate the mutual information of a 2 by 2 table? Could you provide me with a R script to do the MI calculation for a 2 by 2 table?

Is there a formal relationship between positive predictive value and mutual information?

Is it possible to calculate the mutual information for a ROC curve?

A ROC curve is defined by thresholds. Each threshold defines a 2 by 2 table. Can you give me a graph with the MI for each threshold in a ROC curve?

Can you give me a R script to compute MI across thresholds?

Please provide me with R code to estimate MI from score + class labels

I would like to extend this concept to a binary logistic regression setting and the prevalence of the risk factor combinations. Can you give me a R script for a simple logistic regression to calculate its mutual information?

Can you give me a R script for a simple Cox regression to calculate its mutual information?

Please, provide me a comprehensive R script which contains all issues discussed in this session.

You've asked an excellent and deep set of questions! I'll answer them step-by-step, carefully explaining the key ideas and providing the requested R code snippets. At the end, I will also give you a comprehensive R script combining the essential parts.

1️⃣ What is the *information theoretic approach* to individual-level surrogacy?

The **information-theoretic approach** treats individual-level surrogacy from a *predictive* angle, asking:

👉 *How much information about the final outcome can be obtained from the surrogate marker?*

The key quantity is usually **mutual information** between the surrogate and the true outcome.

The *predictive goal* is: given the surrogate, how well can we predict the true outcome at the individual level?

Key reference (from the predictive angle):

> Alonso, A., & Molenberghs, G. (2007). *Surrogacy and information theory*. Biometrical Journal, 49(6), 827-838.
> https://doi.org/10.1002/bimj.200710361

This paper formalizes mutual information for surrogacy assessment!

2️⃣ Difference between *meta-analytic* vs *information-theoretic* approach

Approach	Goal	Typical focus	Metric
**Meta-analytic**	Generalizability across trials	*Trial-level surrogacy*	R^2 at trial level, slope consistency
**Information-theoretic**	Individual prediction	*Individual-level surrogacy*	Mutual Information (MI), likelihood reduction factor

Summary:
Meta-analysis focuses on *external validity* (across studies), whereas Information Theory focuses on *individual-level predictive power* within a study.

3️⃣ What is *mutual information*?

Definition: Mutual information (MI) between two variables X and Y measures how much knowing X reduces uncertainty about Y:

$$I(X;Y) = \sum_{x,y} p(x,y) \log \frac{p(x,y)}{p(x)p(y)}$$

- If MI = 0 → variables are independent.
- Larger MI → more predictive power.

4️⃣ Is there a relationship between *mutual information* and *deviance*?

Yes!
Deviance = −2 × (log-likelihood of model − log-likelihood of saturated model).

MI is related to the difference in log-likelihoods:

$$I(X;Y) = \frac{1}{N} \left[LL_{\text{model with X}} - LL_{\text{null model}} \right]$$

Thus, *likelihood-based deviance reductions can approximate MI* (up to scaling factors).

5️⃣ What is the *Likelihood Reduction Factor* (LRF) and its relation to MI?

LRF is:

$$\text{LRF} = \frac{LL_{\text{model with surrogate}} - LL_{\text{null}}}{LL_{\text{full}} - LL_{\text{null}}}$$

- It expresses how much of the explainable information is captured by the surrogate.
- It is directly interpretable in relation to MI.

6️⃣ How to calculate MI for a 2x2 table? (R script)

For a 2x2 table:

	Y=1	Y=0
X=1	a	b
X=0	c	d

R code:

```r
# Define 2x2 table
table <- matrix(c(50, 10, 5, 35), nrow = 2, byrow = TRUE)
rownames(table) <- c("X=1", "X=0")
colnames(table) <- c("Y=1", "Y=0")

# Convert to probabilities
N <- sum(table)
p_xy <- table / N
p_x <- rowSums(p_xy)
p_y <- colSums(p_xy)

# Calculate MI
MI <- 0
for (i in 1:2) {
  for (j in 1:2) {
    if (p_xy[i,j] > 0) {
      MI <- MI + p_xy[i,j] * log2(p_xy[i,j] / (p_x[i] * p_y[j]))
    }
  }
}
cat("Mutual Information (bits):", MI, "\n")
```

7️⃣ Relationship between *positive predictive value* (PPV) and MI?

No *exact formal* relationship — PPV is a *conditional probability*:

$$\text{PPV} = P(Y = 1 \mid X = 1)$$

Whereas MI is a *global information measure* across the full joint distribution.

However:

- High PPV *can* contribute to high MI if accompanied by strong contrasts across the table.
- But MI captures both PPV and NPV, as well as marginal distributions.

8️⃣ Can you calculate MI for a ROC curve?

Yes!
A ROC curve is based on varying thresholds — each threshold defines a 2x2 table → MI can be computed for each threshold!

9️⃣ Plot MI for each threshold in ROC (R script)

```r
# Example: Simulated scores and binary labels
set.seed(123)
n <- 200
score <- rnorm(n)
label <- ifelse(score + rnorm(n) > 0, 1, 0)

# Create thresholds
thresholds <- seq(min(score), max(score), length.out = 100)
MI_values <- numeric(length(thresholds))

# Function to compute MI for a 2x2 table
compute_mi <- function(tab) {
  N <- sum(tab)
  p_xy <- tab / N
  p_x <- rowSums(p_xy)
  p_y <- colSums(p_xy)
  mi <- 0
  for (i in 1:2) {
    for (j in 1:2) {
      if (p_xy[i,j] > 0) {
        mi <- mi + p_xy[i,j] * log2(p_xy[i,j] / (p_x[i] * p_y[j]))
      }
    }
  }
  return(mi)
}

# Loop over thresholds
for (i in seq_along(thresholds)) {
  pred <- ifelse(score >= thresholds[i], 1, 0)
  tab <- table(pred, label)
  # Pad table if needed
  tab <- as.matrix(tab)
  if (nrow(tab) < 2) tab <- rbind(tab, c(0,0))
  if (ncol(tab) < 2) tab <- cbind(tab, c(0,0))
  MI_values[i] <- compute_mi(tab)
}

# Plot
plot(thresholds, MI_values, type = "l", lwd = 2, col = "blue",
     xlab = "Threshold", ylab = "Mutual Information (bits)",
     main = "MI across ROC thresholds")
```

1️⃣0️⃣ R script: MI from *score* + *class labels*

```r
# Inputs: score and label vectors
score <- rnorm(200)
label <- ifelse(score + rnorm(200) > 0, 1, 0)

# Example threshold = 0
pred <- ifelse(score >= 0, 1, 0)
tab <- table(pred, label)
```

```
# Pad
tab <- as.matrix(tab)
if (nrow(tab) < 2) tab <- rbind(tab, c(0,0))
if (ncol(tab) < 2) tab <- cbind(tab, c(0,0))

# Compute MI
compute_mi(tab)
```

1️⃣1️⃣ MI in *binary logistic regression* — R script

You can compute MI from *likelihood difference* between null and full model:

```r
# Simulate data
set.seed(123)
n <- 200
x <- rbinom(n, 1, 0.5)
y <- rbinom(n, 1, plogis(0.5 * x))

# Logistic regression
model_full <- glm(y ~ x, family = binomial)
model_null <- glm(y ~ 1, family = binomial)

# Compute MI
LL_full <- logLik(model_full)
LL_null <- logLik(model_null)

MI_logreg <- (LL_full - LL_null) / log(2) / n  # Convert to bits per observation
cat("Mutual Information (bits per obs):", MI_logreg, "\n")
```

1️⃣2️⃣ MI in *Cox regression* — R script

Analogous: Use partial likelihood from Cox model.

```r
library(survival)

# Simulate survival data
set.seed(123)
n <- 200
x <- rbinom(n, 1, 0.5)
time <- rexp(n, rate = 0.1 * exp(0.5 * x))
status <- rbinom(n, 1, 0.9)  # censoring indicator

# Cox regression
cox_full <- coxph(Surv(time, status) ~ x)
cox_null <- coxph(Surv(time, status) ~ 1)

# Log-partial likelihood
LL_full <- cox_full$loglik[2]
LL_null <- cox_full$loglik[1]

# MI estimate
MI_cox <- (LL_full - LL_null) / log(2) / n
cat("Mutual Information (bits per obs):", MI_cox, "\n")
```

1️⃣3️⃣ Comprehensive R script (summary of this session)

```r
# ---- MI for 2x2 table ----
compute_mi <- function(tab) {
  N <- sum(tab)
  p_xy <- tab / N
  p_x <- rowSums(p_xy)
  p_y <- colSums(p_xy)
  mi <- 0
  for (i in 1:2) {
    for (j in 1:2) {
      if (p_xy[i,j] > 0) {
        mi <- mi + p_xy[i,j] * log2(p_xy[i,j] / (p_x[i] * p_y[j]))
      }
    }
  }
  return(mi)
}

# Example 2x2 table
table2x2 <- matrix(c(50, 10, 5, 35), nrow = 2, byrow = TRUE)
MI_2x2 <- compute_mi(table2x2)
cat("MI 2x2 table:", MI_2x2, "\n")

# ---- MI across ROC thresholds ----
set.seed(123)
score <- rnorm(200)
label <- ifelse(score + rnorm(200) > 0, 1, 0)

thresholds <- seq(min(score), max(score), length.out = 100)
MI_values <- sapply(thresholds, function(th) {
  pred <- ifelse(score >= th, 1, 0)
  tab <- table(pred, label)
  # Pad
  tab <- as.matrix(tab)
  if (nrow(tab) < 2) tab <- rbind(tab, c(0,0))
  if (ncol(tab) < 2) tab <- cbind(tab, c(0,0))
  compute_mi(tab)
})

plot(thresholds, MI_values, type = "l", lwd = 2, col = "blue",
     xlab = "Threshold", ylab = "Mutual Information (bits)",
     main = "MI across ROC thresholds")

# ---- Logistic regression MI ----
set.seed(123)
x <- rbinom(200, 1, 0.5)
y <- rbinom(200, 1, plogis(0.5 * x))
model_full <- glm(y ~ x, family = binomial)
model_null <- glm(y ~ 1, family = binomial)

LL_full <- logLik(model_full)
LL_null <- logLik(model_null)

MI_logreg <- (LL_full - LL_null) / log(2) / length(y)
cat("MI logistic regression (bits per obs):", MI_logreg, "\n")

# ---- Cox regression MI ----
library(survival)
set.seed(123)
x <- rbinom(200, 1, 0.5)
time <- rexp(200, rate = 0.1 * exp(0.5 * x))
status <- rbinom(200, 1, 0.9)

cox_full <- coxph(Surv(time, status) ~ x)
LL_full <- cox_full$loglik[2]
LL_null <- cox_full$loglik[1]

MI_cox <- (LL_full - LL_null) / log(2) / length(x)
cat("MI Cox regression (bits per obs):", MI_cox, "\n")
```

If you'd like, I can also provide you:

✅ How to *bootstrap MI* for confidence intervals
✅ How to compute MI for *continuous* surrogates
✅ How to *decompose* MI (e.g. in logistic regression models)

Would you like me to generate an even more polished *MI toolbox* in R for you? I can also give you references to more advanced papers on this topic! 🚀
